# Supplementary material for: Oncogenic Properties of the EBV ZEBRA Protein
Source: Cancers (Basel). 2020 Jun 5;12(6):1479. doi: 10.3390/cancers12061479 (PMC7352903; doi:10.3390/cancers12061479)
Supplement: Supplementary file 1 [file cancers-12-01479-s001.pdf]

Supplemental Materials: Oncogenic Properties of the EBV ZEBRA Protein

Diego Germini, Fatimata Bintou Sall, Anna Shmakova, Joelle Wiels, Svetlana Dokudovskaya, Emmanuel Drouet and Yegor Vassetzky

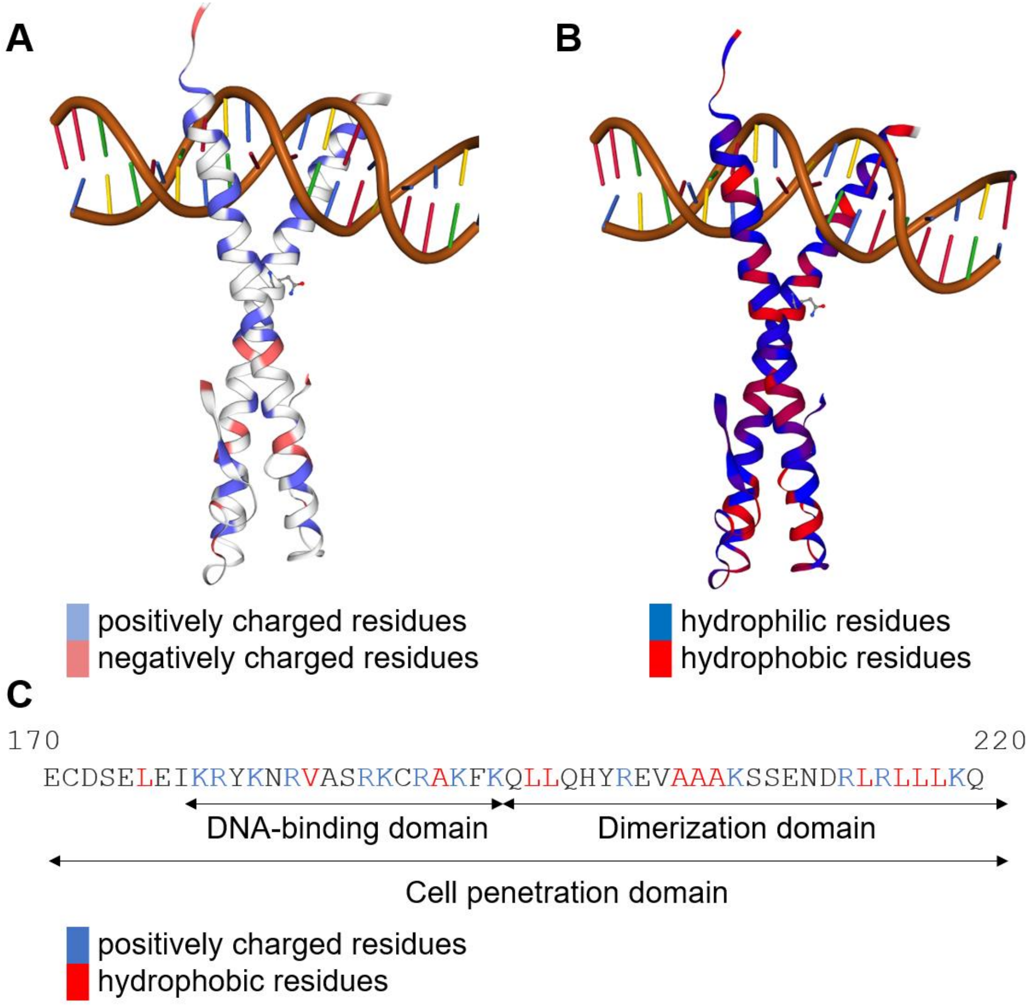

Figure 1. ZEBRA cell penetration domain. **(A)** positions of positively (blue) and negatively (red) charged residues in ZEBRA structure. Residues from 175 (top) to 236 (bottom) were characterized by X-ray crystallography [38] and are available at the SWISS-MODEL Repository [188]. (**B)** the same ZEBRA structure, positions of hydrophilic (blue) and hydrophobic (red) residues are shown. (**C)** primary structure of ZEBRA cell penetration domain (residues 170-220). Positively charged amino acids (seven lysines and seven arginines) are shown in blue, whereas hydrophobic amino acids (seven leucines, five alanines, one valine) are shown in red [35].

© 2020 by the authors. Licensee MDPI, Basel, Switzerland. This article is an open access article distributed under the terms and conditions of the Creative Commons Attribution (CC BY) license (http://creativecommons.org/licenses/by/4.0/).
